# Barium dierbium(III) tetra­sulfide

**DOI:** 10.1107/S1600536813003541

**Published:** 2013-02-13

**Authors:** Adel Mesbah, Wojciech Stojko, James. A Ibers

**Affiliations:** a Department of Chemistry, Northwestern University, 2145 Sheridan Road, Evanston, IL 60208-3113, USA

## Abstract

Barium dierbium(III) tetra­sulfide, BaEr_2_S_4_, crystallizes with four formula units in the ortho­rhom­bic space group *Pnma* in the CaFe_2_O_4_ structure type. The asymmetric unit contains two Er, one Ba, and four S atoms, each with .*m*. site symmetry. The structure consists of channels formed by corner- and edge-sharing ErS_6_ octa­hedra in which Ba atoms reside. The resultant coordination of Ba is that of a bicapped trigonal prism.

## Related literature

The unit-cell parameters of BaEr_2_S_4_, which crystallizes in the CaFe_2_O_4_ structure type (Decker & Kasper, 1957 ▶), were previously determined from X-ray powder diffraction data (Patrie *et al.*, 1964 ▶). For related structures, see: Bugaris & Ibers (2009 ▶); Narducci *et al.* (2000 ▶); Carpenter & Hwu (1992 ▶); Flahaut *et al.* (1965 ▶); Schurz & Schleid (2011 ▶). For synthetic details, see: Bugaris & Ibers (2008 ▶); Haneveld & Jellinek (1969 ▶). For standardization of structural data, see: Gelato & Parthé (1987 ▶).

## Experimental

### 

#### Crystal data

BaEr_2_S_4_
*M* *_r_* = 600.10
Orthorhombic, 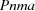

*a* = 12.1455 (3) Å
*b* = 3.9884 (1) Å
*c* = 14.3837 (4) Å
*V* = 696.76 (3) Å^3^
*Z* = 4
Mo *K*α radiation
μ = 30.53 mm^−1^
*T* = 100 K
0.16 × 0.03 × 0.02 mm

#### Data collection

Bruker APEXII CCD diffractometer
Absorption correction: numerical face indexed (Sheldrick, 2008*a* ▶) *T*_min_ = 0.084, *T*_max_ = 0.528
9866 measured reflections
1207 independent reflections
1196 reflections with *I* > 2σ(*I*)
*R*_int_ = 0.030

#### Refinement

*R*[*F* ^2^ > 2σ(*F* ^2^)] = 0.017
*wR*(*F* ^2^) = 0.050
*S* = 1.87
1207 reflections
44 parameters
Δρ_max_ = 2.46 e Å^−3^
Δρ_min_ = −2.58 e Å^−3^

### 

Data collection: *APEX2* (Bruker, 2009 ▶); cell refinement: *SAINT* (Bruker, 2009 ▶); data reduction: *SAINT*; program(s) used to solve structure: *SHELXS97* (Sheldrick, 2008*b* ▶); program(s) used to refine structure: *SHELXL97* (Sheldrick, 2008*b* ▶); molecular graphics: *CrystalMaker* (Palmer, 2012 ▶); software used to prepare material for publication: *SHELXL97*.

## Supplementary Material

Crystal structure: contains datablock(s) I, global. DOI: 10.1107/S1600536813003541/br2222sup1.cif

Structure factors: contains datablock(s) I. DOI: 10.1107/S1600536813003541/br2222Isup2.hkl

Additional supplementary materials:  crystallographic information; 3D view; checkCIF report

## Figures and Tables

**Fig. 1.**
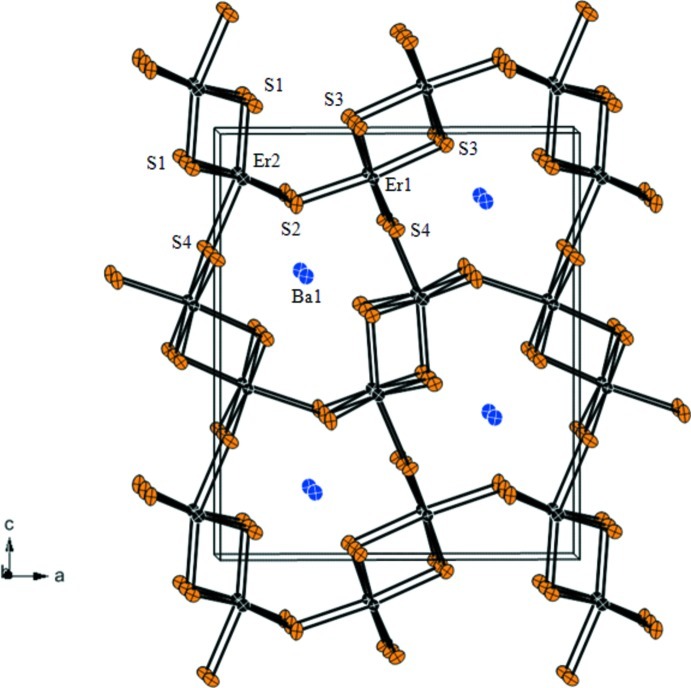
Structure of BaEr2S4 viewed approximatly down [010]. Displacement ellipsoids are drawn at 95% probability level.

## supplementary crystallographic information

### Comment

Orange needles of BaEr_2_S_4_ were obtained in a solid-state reaction. The compound was synthesized previously (Patrie *et al.*, 1964), and its unit cell parameters were determined from X-powder diffraction data. In the BaLn_2_S_4_ family (Ln = rare earth element) no structures have been determined from single-crystal data but that of the closely related compound BaLu_2_S_4_ has (Schurz & Schleid, 2011). Here, from X-ray diffraction single-crystal data we find that BaEr_2_S_4_ crystallizes in the CaFe_2_O_4_ structure type (Decker & Kasper, 1957) with four formula units in space group *Pnma* of the orthorhombic system. In the asymmetric unit there are two Er, one Ba, and four S atoms, each with site symmetry *.m.*. A projection of the structure down [010] is shown in Figure 1. The structure consists of ErS_6_ octahedra that form dimers by edge-sharing. Four such dimers form an infinite channel by corner-sharing in the (010) plane. Each channel is filled by one Ba atom. Each Er1 atom is octahedrally coordinated to one S2, three S3, and two S4 atoms; each Er2 atom is coordinated to three S1, two S2, and one S4 atom. The interatomic Er - S distances at 2.6706 (10) to 2.7376 (7) Å compare favorably to those of 2.672 (4) to 2.720 (4) Å in the structure of BaLu_2_S_4_ (Schurz & Schleid, 2011). As there are no S - S bonds in the structure, formal oxidation states may be assigned as Ba^2+^, Er^3+^, and S^2-^.

### Experimental

In an exploration of the quaternary solid-state Ba/Er/U/S system, orange needles of BaEr_2_S_4_ were obtained instead in a two-step reaction. Uranium powder was obtained by hydridization and decomposition of ^238^U turnings (Oak Ridge National Laboratory) (Bugaris & Ibers, 2008; Haneveld & Jellinek, 1969). The other reactants were used as obtained. In the first step, a mixture consisting of powdered ^238^U (20.9 mg, 0.088 mmol), Er (14.0 mg, 0.084 mmol), BaS (42.7 mg, 0.252 mmol), and S (8.0 mg, 0.25 mmol) was loaded into a carbon-coated fused-silica tube under an Ar atmosphere in a glove box. The tube was evacuated to 10 ^-4^ Torr, and flame sealed. It was placed in computer-contolled furnace, heated to 1273 K in 48 h, held there for 8 d, then cooled to 293 K at 3 K/h. In the second step, the resultant black powder was ground and mixed thoroughly with 50 mg of Sb_2_S_3_. This mixture was re-loaded into a carbon-coated fused-silica tube, evacuated, sealed, and then placed in a computer-controlled furnace The tube was heated to 1273 K in 24 h, held there for 4 d, then cooled to 293 K at 2 K/h. Orange needles were obtained in about 50 wt% yield. Analysis of these orange crystals on an EDX– equipped Hitachi S-3400 SEM showed the presence of Ba, Er, and S in the approximate ratio 1:2:4 but no U. The other products were black crystals of Sb_2_S_3_ and US_2_.

### Refinement

The structure was standardized by means of the program *STRUCTURE TIDY* ((Gelato & Parthé, 1987). The highest peak in the difference electron density map (2.46 e. Å^-3^) was 0.47 Å from atom Er1 and the deepest hole (- 2.58 e. Å^-3^) was 0.20 Å from atom Ba1.

### Crystal data

**Table:**

BaEr_2_S_4_	*F*(000) = 1024
*M**_r_* = 600.10	*D*_x_ = 5.721 Mg m^−^^3^
Orthorhombic, *P**n**m**a*	Mo *K*α radiation, λ = 0.71073 Å
Hall symbol: -P 2ac 2n	Cell parameters from 8150 reflections
*a* = 12.1455 (3) Å	θ = 2.3–30.5°
*b* = 3.9884 (1) Å	µ = 30.53 mm^−^^1^
*c* = 14.3837 (4) Å	*T* = 100 K
*V* = 696.76 (3) Å^3^	Needle, orange
*Z* = 4	0.16 × 0.03 × 0.02 mm

### Data collection

**Table:**

Bruker APEXII CCD diffractometer	1207 independent reflections
Radiation source: fine-focus sealed tube	1196 reflections with *I* > 2σ(*I*)
Graphite monochromator	*R*_int_ = 0.030
φ and ω scans	θ_max_ = 30.5°, θ_min_ = 2.2°
Absorption correction: numerical face indexed (Sheldrick, 2008*a*)	*h* = −17→17
*T*_min_ = 0.084, *T*_max_ = 0.528	*k* = −5→5
9866 measured reflections	*l* = −20→20

### Refinement

**Table:**

Refinement on *F*^2^	Primary atom site location: structure-invariant direct methods
Least-squares matrix: full	Secondary atom site location: difference Fourier map
*R*[*F*^2^ > 2σ(*F*^2^)] = 0.017	1/[s^2^(*F*_o_^2^) + (0.0192*F*_o_^2^)^2^]
*wR*(*F*^2^) = 0.050	(Δ/σ)_max_ = 0.002
*S* = 1.87	Δρ_max_ = 2.46 e Å^−^^3^
1207 reflections	Δρ_min_ = −2.58 e Å^−^^3^
44 parameters	Extinction correction: *SHELXL97* (Sheldrick, 2008a), Fc^*^=kFc[1+0.001xFc^2^λ^3^/sin(2θ)]^-1/4^
0 restraints	Extinction coefficient: 0.00257 (16)

### Fractional atomic coordinates and isotropic or equivalent isotropic displacement parameters (Å^2^)

**Table:**

	*x*	*y*	*z*	*U*_iso_*/*U*_eq_	
Er1	0.079448 (16)	0.2500	0.398817 (12)	0.00349 (8)	
Er2	0.566211 (16)	0.2500	0.608465 (12)	0.00444 (8)	
Ba1	0.24191 (2)	0.2500	0.662667 (18)	0.00705 (9)	
S1	0.08229 (8)	0.2500	0.07671 (7)	0.00485 (18)	
S2	0.29294 (9)	0.2500	0.33808 (7)	0.00537 (18)	
S3	0.37564 (8)	0.2500	0.02341 (7)	0.00449 (18)	
S4	0.47727 (8)	0.2500	0.78311 (7)	0.00490 (18)	

### Atomic displacement parameters (Å^2^)

**Table:**

	*U*^11^	*U*^22^	*U*^33^	*U*^12^	*U*^13^	*U*^23^
Er1	0.00359 (11)	0.00300 (12)	0.00388 (11)	0.000	0.00010 (5)	0.000
Er2	0.00495 (12)	0.00349 (12)	0.00487 (11)	0.000	−0.00020 (5)	0.000
Ba1	0.00595 (14)	0.00835 (14)	0.00683 (13)	0.000	0.00015 (8)	0.000
S1	0.0057 (4)	0.0040 (4)	0.0049 (4)	0.000	−0.0004 (3)	0.000
S2	0.0037 (4)	0.0044 (4)	0.0080 (4)	0.000	−0.0006 (3)	0.000
S3	0.0046 (4)	0.0037 (4)	0.0052 (4)	0.000	0.0009 (3)	0.000
S4	0.0061 (4)	0.0039 (4)	0.0047 (4)	0.000	0.0015 (3)	0.000

### Geometric parameters (Å, º)

**Table:**

Er1—S4^i^	2.6872 (6)	Ba1—S4	3.3426 (11)
Er1—S4^ii^	2.6872 (6)	Ba1—Er2^x^	3.9231 (3)
Er1—S3^iii^	2.7164 (10)	Ba1—Ba1^xi^	3.9884 (1)
Er1—S3^iv^	2.7360 (7)	Ba1—Ba1^xii^	3.9884
Er1—S3^v^	2.7360 (7)	S1—Er2^iii^	2.6706 (10)
Er1—S2	2.7362 (11)	S1—Er2^i^	2.7273 (7)
Er1—Ba1	4.2774 (3)	S1—Er2^ii^	2.7273 (7)
Er2—S1^vi^	2.6706 (10)	S1—Ba1^ii^	3.1725 (8)
Er2—S1^v^	2.7274 (7)	S1—Ba1^i^	3.1725 (8)
Er2—S1^iv^	2.7274 (7)	S2—Er2^vii^	2.7376 (7)
Er2—S4	2.7345 (9)	S2—Er2^viii^	2.7376 (7)
Er2—S2^vii^	2.7376 (7)	S2—Ba1^i^	3.2437 (9)
Er2—S2^viii^	2.7376 (7)	S2—Ba1^ii^	3.2437 (9)
Er2—Ba1^ix^	3.9231 (3)	S3—Er1^vi^	2.7163 (10)
Er2—Ba1	4.0153 (3)	S3—Er1^i^	2.7360 (7)
Ba1—S3^v^	3.1666 (8)	S3—Er1^ii^	2.7360 (7)
Ba1—S3^iv^	3.1666 (8)	S3—Ba1^ii^	3.1666 (8)
Ba1—S1^iv^	3.1725 (8)	S3—Ba1^i^	3.1666 (8)
Ba1—S1^v^	3.1725 (8)	S4—Er1^iv^	2.6873 (6)
Ba1—S2^iv^	3.2438 (9)	S4—Er1^v^	2.6873 (6)
Ba1—S2^v^	3.2438 (9)	S4—Ba1^ix^	3.3074 (11)
Ba1—S4^x^	3.3074 (11)		
			
S4^i^—Er1—S4^ii^	95.82 (3)	S3^iv^—Ba1—Er2^x^	106.592 (19)
S4^i^—Er1—S3^iii^	91.23 (3)	S1^iv^—Ba1—Er2^x^	133.879 (16)
S4^ii^—Er1—S3^iii^	91.23 (3)	S1^v^—Ba1—Er2^x^	133.879 (16)
S4^i^—Er1—S3^iv^	176.07 (3)	S2^iv^—Ba1—Er2^x^	43.642 (15)
S4^ii^—Er1—S3^iv^	85.17 (2)	S2^v^—Ba1—Er2^x^	43.642 (15)
S3^iii^—Er1—S3^iv^	84.95 (3)	S4^x^—Ba1—Er2^x^	43.408 (16)
S4^i^—Er1—S3^v^	85.17 (2)	S4—Ba1—Er2^x^	91.735 (18)
S4^ii^—Er1—S3^v^	176.07 (3)	S3^v^—Ba1—Ba1^xi^	129.032 (11)
S3^iii^—Er1—S3^v^	84.95 (3)	S3^iv^—Ba1—Ba1^xi^	50.967 (11)
S3^iv^—Er1—S3^v^	93.58 (3)	S1^iv^—Ba1—Ba1^xi^	51.054 (12)
S4^i^—Er1—S2	92.59 (3)	S1^v^—Ba1—Ba1^xi^	128.946 (12)
S4^ii^—Er1—S2	92.59 (3)	S2^iv^—Ba1—Ba1^xi^	52.064 (12)
S3^iii^—Er1—S2	174.30 (3)	S2^v^—Ba1—Ba1^xi^	127.936 (12)
S3^iv^—Er1—S2	91.16 (3)	S4^x^—Ba1—Ba1^xi^	90.0
S3^v^—Er1—S2	91.16 (3)	S4—Ba1—Ba1^xi^	90.0
S4^i^—Er1—Ba1	131.894 (15)	Er2^x^—Ba1—Ba1^xi^	90.0
S4^ii^—Er1—Ba1	131.894 (15)	S3^v^—Ba1—Ba1^xii^	50.967 (11)
S3^iii^—Er1—Ba1	93.15 (2)	S3^iv^—Ba1—Ba1^xii^	129.032 (11)
S3^iv^—Er1—Ba1	47.693 (15)	S1^iv^—Ba1—Ba1^xii^	128.946 (12)
S3^v^—Er1—Ba1	47.693 (15)	S1^v^—Ba1—Ba1^xii^	51.054 (12)
S2—Er1—Ba1	81.15 (2)	S2^iv^—Ba1—Ba1^xii^	127.936 (12)
S1^vi^—Er2—S1^v^	83.18 (3)	S2^v^—Ba1—Ba1^xii^	52.064 (12)
S1^vi^—Er2—S1^iv^	83.18 (3)	S4^x^—Ba1—Ba1^xii^	90.0
S1^v^—Er2—S1^iv^	93.97 (3)	S4—Ba1—Ba1^xii^	90.0
S1^vi^—Er2—S4	160.92 (3)	Er2^x^—Ba1—Ba1^xii^	90.0
S1^v^—Er2—S4	83.84 (3)	Ba1^xi^—Ba1—Ba1^xii^	180.0
S1^iv^—Er2—S4	83.84 (3)	S3^v^—Ba1—Er2	108.630 (19)
S1^vi^—Er2—S2^vii^	103.56 (3)	S3^iv^—Ba1—Er2	108.630 (19)
S1^v^—Er2—S2^vii^	173.17 (3)	S1^iv^—Ba1—Er2	42.616 (14)
S1^iv^—Er2—S2^vii^	85.85 (2)	S1^v^—Ba1—Er2	42.616 (14)
S4—Er2—S2^vii^	89.36 (3)	S2^iv^—Ba1—Er2	106.201 (19)
S1^vi^—Er2—S2^viii^	103.56 (3)	S2^v^—Ba1—Er2	106.201 (19)
S1^v^—Er2—S2^viii^	85.85 (2)	S4^x^—Ba1—Er2	177.557 (18)
S1^iv^—Er2—S2^viii^	173.17 (3)	S4—Ba1—Er2	42.413 (17)
S4—Er2—S2^viii^	89.36 (3)	Er2^x^—Ba1—Er2	134.149 (8)
S2^vii^—Er2—S2^viii^	93.51 (3)	Ba1^xi^—Ba1—Er2	90.0
S1^vi^—Er2—Ba1^ix^	142.85 (2)	Ba1^xii^—Ba1—Er2	90.0
S1^v^—Er2—Ba1^ix^	120.02 (2)	Er2^iii^—S1—Er2^i^	96.82 (3)
S1^iv^—Er2—Ba1^ix^	120.02 (2)	Er2^iii^—S1—Er2^ii^	96.82 (3)
S4—Er2—Ba1^ix^	56.22 (2)	Er2^i^—S1—Er2^ii^	93.97 (3)
S2^vii^—Er2—Ba1^ix^	54.861 (19)	Er2^iii^—S1—Ba1^ii^	115.97 (3)
S2^viii^—Er2—Ba1^ix^	54.861 (19)	Er2^i^—S1—Ba1^ii^	147.08 (4)
S1^vi^—Er2—Ba1	105.39 (2)	Er2^ii^—S1—Ba1^ii^	85.422 (12)
S1^v^—Er2—Ba1	51.961 (18)	Er2^iii^—S1—Ba1^i^	115.97 (3)
S1^iv^—Er2—Ba1	51.961 (18)	Er2^i^—S1—Ba1^i^	85.422 (12)
S4—Er2—Ba1	55.53 (2)	Er2^ii^—S1—Ba1^i^	147.08 (4)
S2^vii^—Er2—Ba1	123.95 (2)	Ba1^ii^—S1—Ba1^i^	77.89 (2)
S2^viii^—Er2—Ba1	123.95 (2)	Er1—S2—Er2^vii^	120.17 (3)
Ba1^ix^—Er2—Ba1	111.755 (5)	Er1—S2—Er2^viii^	120.17 (3)
S3^v^—Ba1—S3^iv^	78.06 (2)	Er2^vii^—S2—Er2^viii^	93.51 (3)
S3^v^—Ba1—S1^iv^	116.92 (2)	Er1—S2—Ba1^i^	97.16 (3)
S3^iv^—Ba1—S1^iv^	70.19 (2)	Er2^vii^—S2—Ba1^i^	138.40 (4)
S3^v^—Ba1—S1^v^	70.19 (2)	Er2^viii^—S2—Ba1^i^	81.498 (14)
S3^iv^—Ba1—S1^v^	116.92 (2)	Er1—S2—Ba1^ii^	97.16 (3)
S1^iv^—Ba1—S1^v^	77.89 (2)	Er2^vii^—S2—Ba1^ii^	81.498 (14)
S3^v^—Ba1—S2^iv^	145.12 (3)	Er2^viii^—S2—Ba1^ii^	138.40 (4)
S3^iv^—Ba1—S2^iv^	92.639 (19)	Ba1^i^—S2—Ba1^ii^	75.87 (2)
S1^iv^—Ba1—S2^iv^	90.26 (2)	Er1^vi^—S3—Er1^i^	95.05 (3)
S1^v^—Ba1—S2^iv^	141.02 (3)	Er1^vi^—S3—Er1^ii^	95.05 (3)
S3^v^—Ba1—S2^v^	92.639 (19)	Er1^i^—S3—Er1^ii^	93.58 (3)
S3^iv^—Ba1—S2^v^	145.12 (3)	Er1^vi^—S3—Ba1^ii^	98.65 (3)
S1^iv^—Ba1—S2^v^	141.02 (3)	Er1^i^—S3—Ba1^ii^	164.41 (4)
S1^v^—Ba1—S2^v^	90.26 (2)	Er1^ii^—S3—Ba1^ii^	92.590 (8)
S2^iv^—Ba1—S2^v^	75.87 (2)	Er1^vi^—S3—Ba1^i^	98.65 (3)
S3^v^—Ba1—S4^x^	73.20 (2)	Er1^i^—S3—Ba1^i^	92.590 (8)
S3^iv^—Ba1—S4^x^	73.20 (2)	Er1^ii^—S3—Ba1^i^	164.41 (4)
S1^iv^—Ba1—S4^x^	138.242 (15)	Ba1^ii^—S3—Ba1^i^	78.07 (2)
S1^v^—Ba1—S4^x^	138.242 (15)	Er1^iv^—S4—Er1^v^	95.82 (3)
S2^iv^—Ba1—S4^x^	71.93 (2)	Er1^iv^—S4—Er2	132.085 (15)
S2^v^—Ba1—S4^x^	71.93 (2)	Er1^v^—S4—Er2	132.085 (15)
S3^v^—Ba1—S4	135.514 (16)	Er1^iv^—S4—Ba1^ix^	95.91 (3)
S3^iv^—Ba1—S4	135.514 (16)	Er1^v^—S4—Ba1^ix^	95.91 (3)
S1^iv^—Ba1—S4	68.06 (2)	Er2—S4—Ba1^ix^	80.37 (3)
S1^v^—Ba1—S4	68.06 (2)	Er1^iv^—S4—Ba1	95.84 (3)
S2^iv^—Ba1—S4	73.05 (2)	Er1^v^—S4—Ba1	95.84 (3)
S2^v^—Ba1—S4	73.05 (2)	Er2—S4—Ba1	82.05 (3)
S4^x^—Ba1—S4	135.144 (10)	Ba1^ix^—S4—Ba1	162.42 (3)
S3^v^—Ba1—Er2^x^	106.592 (19)		

Symmetry codes: (i) −*x*+1/2, −*y*, *z*−1/2; (ii) −*x*+1/2, −*y*+1, *z*−1/2; (iii) *x*−1/2, *y*, −*z*+1/2; (iv) −*x*+1/2, −*y*+1, *z*+1/2; (v) −*x*+1/2, −*y*, *z*+1/2; (vi) *x*+1/2, *y*, −*z*+1/2; (vii) −*x*+1, −*y*+1, −*z*+1; (viii) −*x*+1, −*y*, −*z*+1; (ix) *x*+1/2, *y*, −*z*+3/2; (x) *x*−1/2, *y*, −*z*+3/2; (xi) *x*, *y*+1, *z*; (xii) *x*, *y*−1, *z*.
